# Simultaneous realization of high sensing sensitivity and tunability in plasmonic nanostructures arrays

**DOI:** 10.1038/s41598-017-17024-7

**Published:** 2017-12-01

**Authors:** Yuan-Fong Chou Chau, Chan-Kuang Wang, Linfang Shen, Chee Ming Lim, Hai-Pang Chiang, Chung-Ting Chou Chao, Hung Ji Huang, Chun-Ting Lin, N. T. R. N. Kumara, Nyuk Yoong Voo

**Affiliations:** 1 Centre for Advanced Material and Energy Sciences, Universiti Brunei Darussalam, Tungku Link, Gadong, BE1410 Negara Brunei Darussalam; 2 Department of Electronic Engineering, Chien Hsin University of Science and Technology, No. 229, Jianxing Rd., Zhongli City, Taoyuan County 32097 Taiwan (R.O.C.); 3 Institute of Space Science and Technology, Nanchang University, Nanchang, 330031 China; 4 Institute of Optoelectronic Sciences, National Taiwan Ocean University, No. 2 Pei-Ning Rd., 202, Keelung, Taiwan; 5 Institute of Physics, Academia Sinica, Taipei, Taiwan; 6 Department of Physics, Fu Jen Catholic University, New Taipei City, Taiwan; 7 Instrument Technology Research Center, National Applied Research Laboratories, Hsinchu, Taiwan

## Abstract

A plasmonic nanostructure (PNS) which integrates metallic and dielectric media within a single structure has been shown to exhibit specific plasmonic properties which are considered useful in refractive index (RI) sensor applications. In this paper, the simultaneous realization of sensitivity and tunability of the optical properties of PNSs consisting of alternative Ag and dielectric of nanosphere/nanorod array have been proposed and compared by using three-dimensional finite element method. The proposed system can support plasmonic hybrid modes and the localized surface plasmonic resonances and cavity plasmonic resonances within the individual PNS can be excited by the incident light. The proposed PNSs can be operated as RI sensor with a sensitivity of 500 nm/RIU (RIU = refractive index unit) ranging from UV to the near-infrared. In addition, a narrow bandwidth and nearly zero transmittance along with a high absorptance can be achieved by a denser PNSs configuration in the unit cell of PNS arrays. We have demonstrated the number of modes sustained in the PNS system, as well as, the near-field distribution can be tailored by the dielectric media in PNSs.

## Introduction

Surface plasmon resonance (SPR) produced by the collective oscillations of free electrons at resonance wavelength (λ_res_) occurs in plasmonic nanoparticles (NPs) when these NPs are exposed to light from the ultraviolet (UV) to the near-infrared^1–10^. In the various studies to find applications of SPR, metal nanoparticle (MNP) arrays have been shown to have diverse applications because of their ability to give rise to enhanced local electromagnetic (EM) waves within a sub-wavelength nanometer-sized scale^11–17^. In periodic MNP configuration, the SPRs can be excited at the interface between the MNPs and the dielectric media, when the energy and momentum of the incident light match that of the SP waves^18–22^. The application of MNP array is a rapidly developing area, and the use of MNP arrays have been demonstrated in fields ranging from information processing, communication to quantum optics and bioscience^23–29^.

Plasmonic nanostructures (PNSs) are used to describe the combination of structured metallic and dielectric media formed within a single structure. This combination is often found to be useful because light can localized at the metal-dielectric interfaces. The localization of light exhibits many novel properties that are unattainable in nanostructures of a single material^26^. One important novel property found in metal-dielectric combinations is the strong plasmon-exciton coupling, and the nonlinear optical response can, when combined, produces new physical behaviors on one hand, and in the other hand gives more controlled capabilities via the tuning of the dielectric medium of the PNSs. The tuning occurs through the manipulation of the SPs by the changing of the dielectric environment^1,2,14–16^.

It is well known that refractive index (RI) sensors having extraordinarily high spectral sensitivity can be achieved using SPR modes^11,16,19,21,23,24,30^. The enhancement and confinement of light by plasmonics allows a high density of independent subwavelength sensor elements to be formed in the periodic MNP arrays of RI sensors. The excitation of SPs in periodic MNP arrays produce many outstanding optical properties, such as manifest E-field enhancement, ultra-sensitivity SPR, and propagation at the nanometer-sized region^31^. In sensor application, one would make use of the RI-induced excessively variant E-field accumulation in/on the surface of PNSs which response to chemical modification of the dielectric cores in the PNSs, and with molecular-selective adsorbents this could easily be used in sensing applications^27^.

The recent interests in SPR RI sensors have resulted in works leading to high sensitivity detection technology, however, many of the works have neglected the tunability of the sensor for performance enhancement. The tunability of RI sensors have to be considered because these sensors are expected to be used across a wide working wavelengths^1,19,30,32–34^. The simultaneous realization of high sensitivity and tunability in PNSs with respect to the EM field enhancement and absorption of light in the subwavelength dimensions is still a challenge. The main design strategy is to harmonically couple the localized SPR and the cavity plasmon resonance (CPR) modes. The SPR and CPR modes in these PNSs generate near-field intensity peaks and transmittance dips near the phase-matching conditions. This phenomenon is caused by the hybridization plasmon effects^29,35–37^. It is, therefore, essential to investigate the properties of such PNSs to understand how the PNSs determine their specific plasmon-related properties.

Due to the diverse dissimilarity of sensing purposes, correct sensing structures should be selected simultaneously for high tunability, sensitivity and biocompatibility. Among the different aspects of MNPs, Ag or Au nanospheres/nanorods are commonly used in plasmonic field generation^13,16,25^. In this paper, two types of PNS configurations based on the combination of alternative Ag and dielectric medium of nanosphere/nanorod arrays have been proposed and compared by using the three-dimensional (3-D) finite element method (FEM). We have demonstrated the number of modes sustained in the PNS system, as well as, the near-field distribution can be tailored by the dielectric media in PNSs. In such PNS systems, the light energy is efficiently trapped in the plane of the PNS array and/or the dielectric core of the PNSs, and the trapped light can be utilized for further reactions. By appropriate tailoring of the structure and material parameters, the specific sensing schematics provided in this paper can be implemented to fit the purpose of specific sensor.

## Results

**Figure 1:**
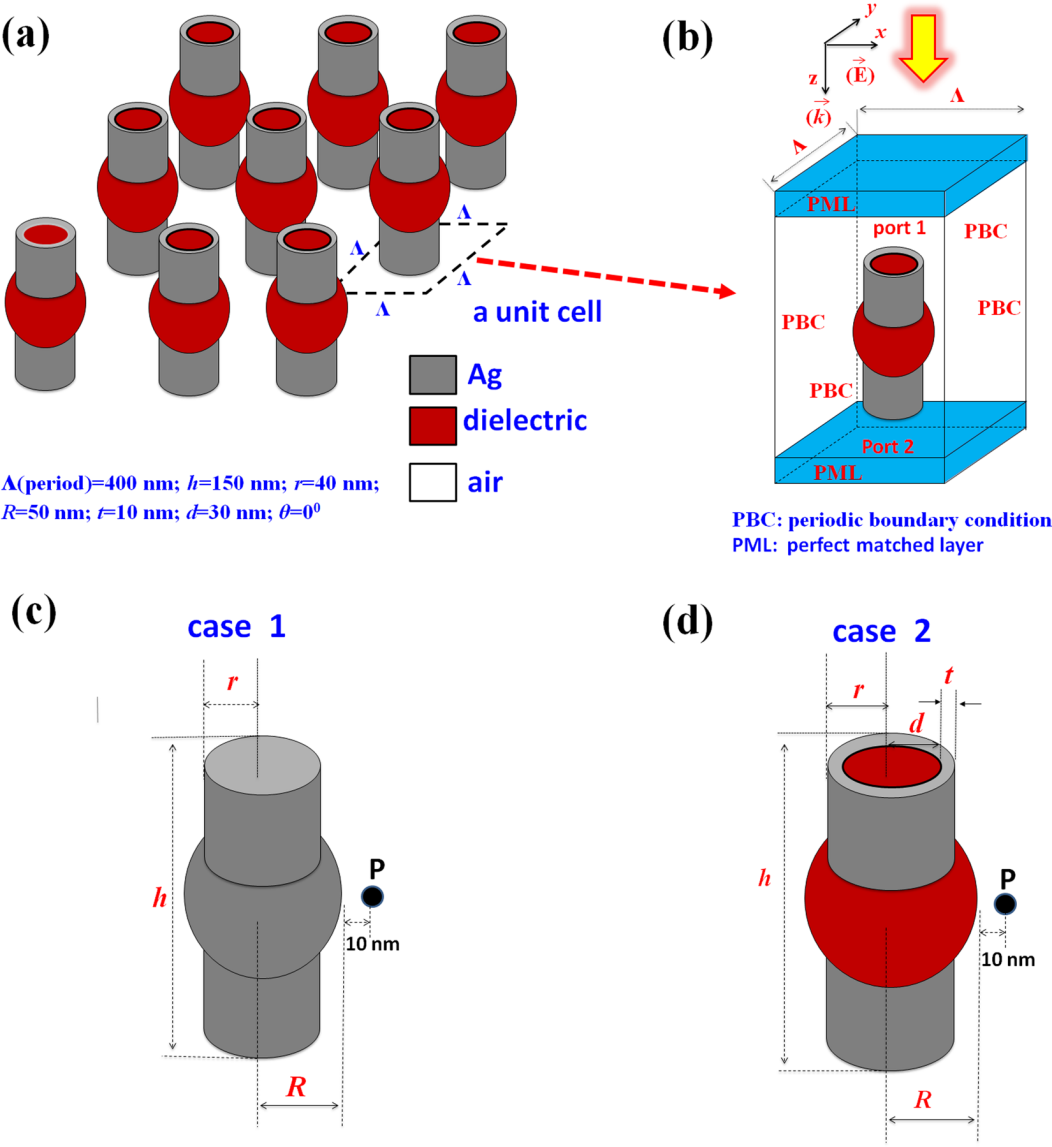
(**a**) The truncated view of a 2-D periodic array of PNSs. (**b**) The unit cell of the two proposed cases, i.e., (**c**) case 1: an all-metal PNS with a combination of a metal nanosphere and a metal nanorod and (**d**) case 2: a combination of a metal nanosphere and a core-shell nanorod (metal-shell with a dielectric nanorod in core region), respectively. The axis shows the propagation direction and polarization of the incident EM wave where the period, height of the nanorod, radius of the nanorod, radius of the nanophere, thickness of the Ag nanoshell and radius of the dielectric nanorod in Ag nanoshell are denoted by *Λ, h, r, R, t* and *d*, respectively. The environmental refractive index is set to be *n* = 1.0 for air.

The PNSs investigated in this work are the combinations of alternative metallic and dielectric nanosphere/nanorod arrays. Figure 1a is the truncated view of a two-dimensional array of PNSs, and the schematic diagram of the unit cell is shown in Fig. 1b. The proposed two cases of unit cells are shown in Fig. 1c and d. Case 1: an all-metal PNS with a combination of a metal nanosphere and a metal nanorod, and case 2: a combination of a metal nanosphere and a core-shell nanorod (metal-shell with a dielectric nanorod in the core region). The value of the incident EM wave is fixed at |***E***_**0**_| = 1 V/m and normally incident from the top surface of the structure. The parameters used in this work are *Λ* (period along the *x* and *y* axes) = 400 nm, *h* (height of the nanorod) = 150 nm, *r* (radius of the nanorod) = 40 nm, *R* (radius of the nanosphere) = 50 nm, *t* (thickness of the Ag nanoshell) = 10 nm, and *d* (radius of the nanorod in Ag nanoshell) = 30 nm, respectively, throughout this paper otherwise specified, and the environmental RI is set at *n* = 1.0 for air. The origin ((x, y, z) = (0, 0, 0)) of the coordinate system is positioned in the middle plane of the simulation zone.

The rapid development of fabrication technology is helping the proposed PNSs to be constructed, especially the use of secondary electron lithography by ion beam milling^38–41^. There are techniques to produce excellent shell-to-shell uniformity^13,37^ such as spacer lithography which can produce uniformly patterned nanoshell with sub-10nm thickness (e.g., *t* from 5 to 10 nm) at the wafer scale^40,41^.

**Figure 2:**
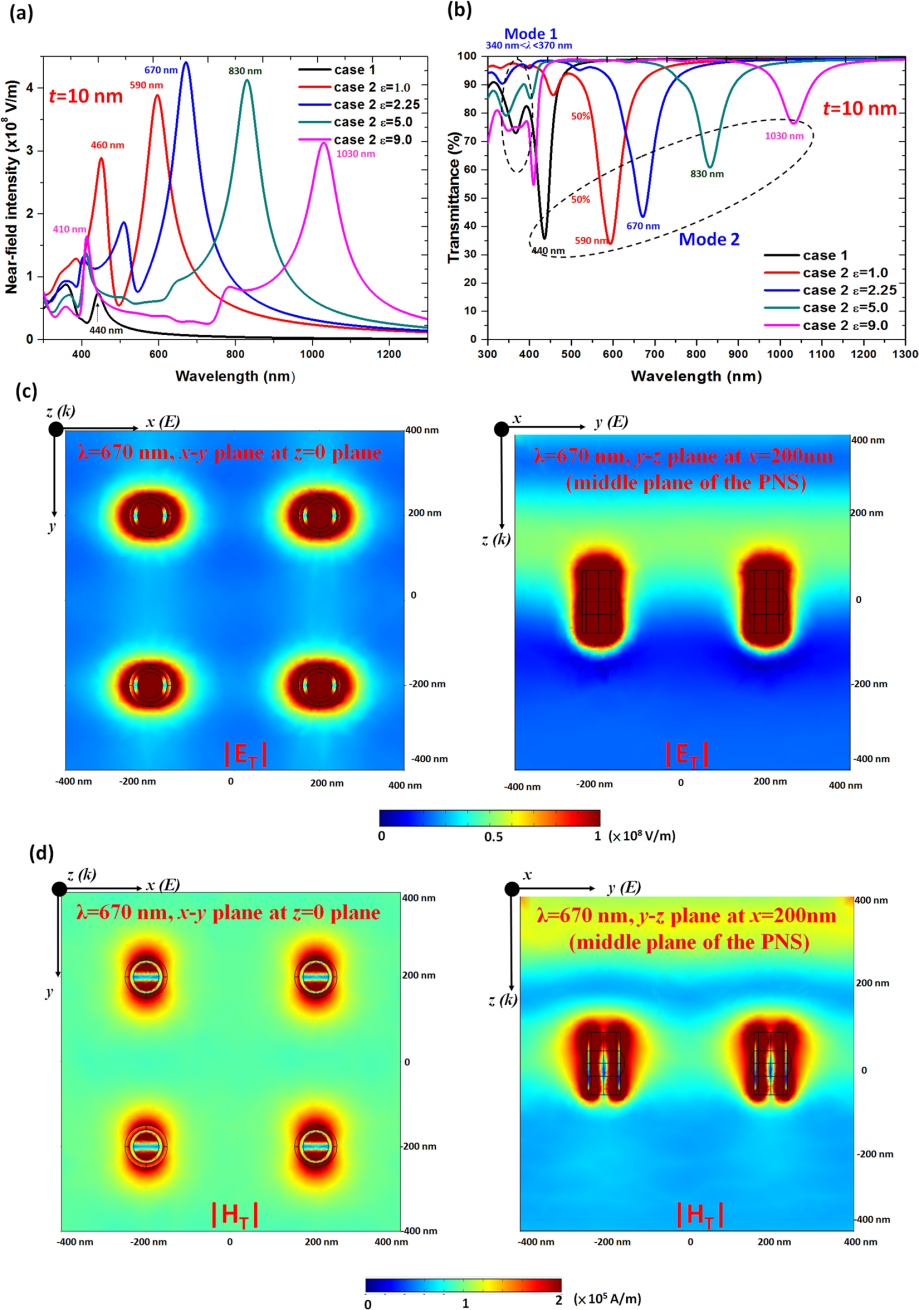
(**a**) Near-field intensity and (**b**) transmittance spectra of case 2 with tuning ε (ε = 1.0, 2.25, 5.0 and 9.0, where ε is the permittivity of dielectric media in PNSs). Case 1 is also displayed for comparison. (**c**) Distributions of the electric field intensity |***E***_***T***_| (in the *x*-y and *x*-*z* plane), and (**d**) Magnetic field intensity |***H***_***T***_| (in the *x*-*y* and *x*-*z* plane) of case 2 (with *ε* *=* 2.25 at λ_res_ = 670 nm).

Figure 2a and b show the near-field intensity and transmittance spectra of case 2 with tuning ε (ε = 1.0, 2.25, 5.0 and 9.0, where ε is the permittivity of dielectric media in PNSs), and case 1 is also displayed to aid the comparison. As observed, the formation of the Ag-shell nanorod with inner and outer dielectric media (*ε*) in case 2 caused the broadband to develop into two distinct modes; mode 1 in 340 < λ_res_ < 370 nm and mode 2 in 440 < λ_res_ < 1030 nm. To attenuate the signal loss in biological imaging and sensing, SP of λ_res_ in the biological window (650–1150 nm) is preferred. The discrepancy of the near-field intensity and transmittance spectra between case 1 and case 2 can be attributed to the geometrical structure of Ag-shell/dielectric-core nanorod in case 2. This feature indicates that the significant Fabry-Perot oscillations and more perpendicular CPR effects exist in case 2 when compared to case 1. To explain the physical mechanism, the electric field (|***E***_***T***_|) and magnetic field (|***H***_***T***_|) of a selected case 2 with *ε* *=* 2.25 at λ_res_ = 670 nm in both the *x*-*y* and *x*-*z* planes are calculated (see Fig. 2c and d and the colorbars are linear scale throughout this paper). It is clear that both the |E_T_| and |H_T_| are effectively localized on the inner and outer surfaces of the PNSs, and show a bonding mode resonance along with a high absorption in PNSs (see the supplementary document). With the help of the Ag-shell nanorod film in case 2, the SPRs and CPRs can be well excited since the |E_T_| and |H_T_| are strongly enhanced inner and outer surface of the PNSs along with an edge enhancement profile, and only a small part of the |E_T_| and |H_T_| runs away to the outside.

**Figure 3:**
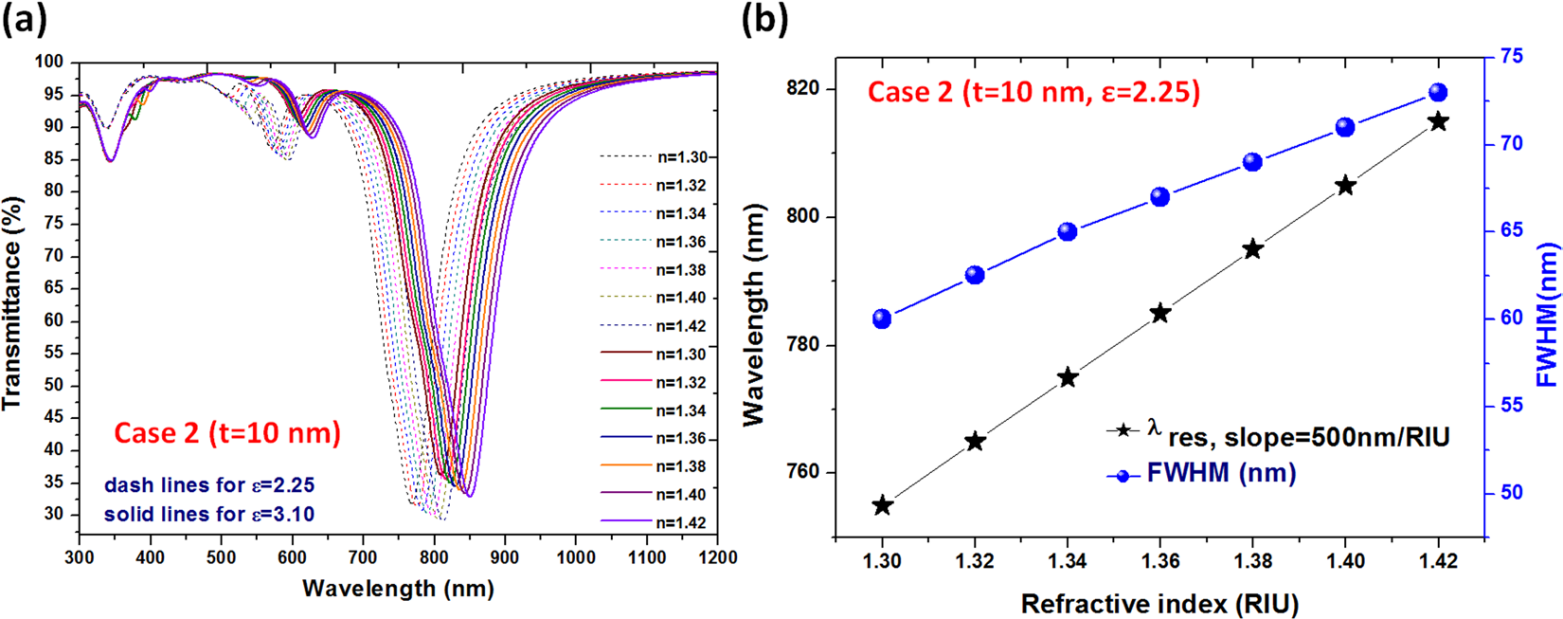
(**a**) Transmittance spectrum of case 2 (with ε = 2.25 (dashed lines) and 3.1 (solid lines)) in the index-matching liquid from *n* = 1.30 to *n* = 1.42 RIU (RIU = refractive index unit), where *n* is the surrounding medium of PNSs; and (**b**) Relation between the wavelength shift and the full width at half maximum (FWHM) versus RI variation.

To investigate the influence of these effects on the performance of the proposed sensor, Fig. 3a shows the transmission spectra of a selected mode 2 of case 2 (with ε = 2.25 and ε = 3.1) in different index-matching liquids, where refractive index unit (RIU) *n* is varied from 1.300 to 1.420 (where *n* is the surrounding medium of PNSs). The dips are caused by the coupling of energy to the SP wave in proximity to the PNSs at λ_res_. With the increase of RI, a red shift of the transmittance dip is observed, and the shift is from 755 to 815 nm for the case of ε = 2.25 (dashed lines) and from 805 to 855 nm (solid lines) for the case of ε = 3.1. The depth and position of transmittance dips can be tuned by varying ε in the PNSs, when the surrounding medium (i.e., refractive index *n*) is introduced in the PNS system. Figure 3b depicts the linear relation between the Δλ (wavelength shifts), and the full width at half maximum (FWHM) versus RI variation in the case of ε = 2.25. The slope of the curves represents the sensitivity. With the increase of RI, the sensitivity raised to a value of 500 nm/RIU. Therefore, the simultaneous realization of tunable and sensitive transmission dip obtained from case 2 exhibits an obvious red-shift as the ambient RI increases. This feature provides a remarkable scheme of nanoscale sensing for the RI variations of the environmental dielectrics such as applications in liquid and gas sensing.

**Figure 4:**
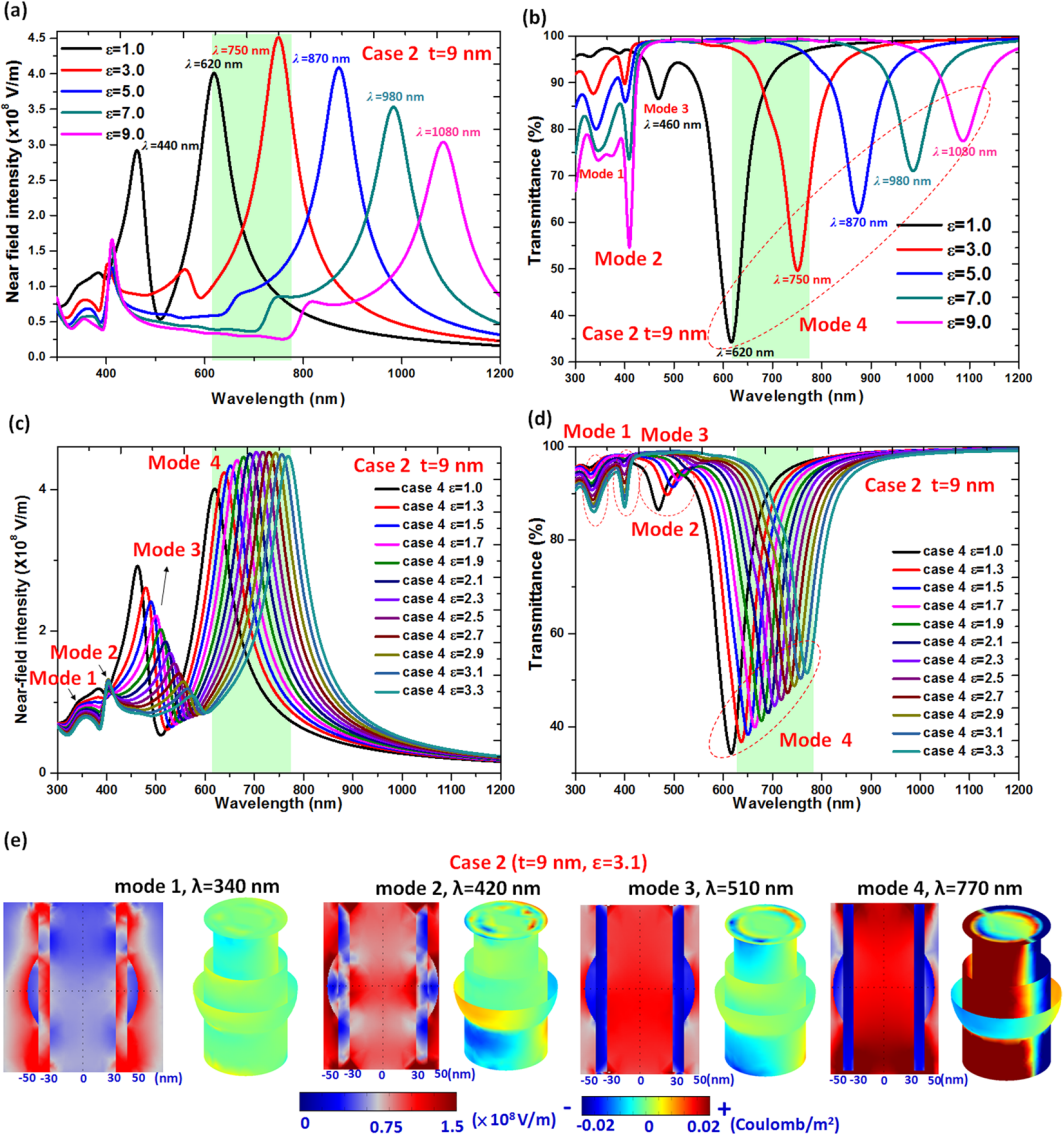
(**a**) Near-field intensity and (**b**) transmittance spectra of case 2 with *t* = 9 nm and varied ε (*ε* = 1.0, 3.0, 5.0, 7.0 and 9.0, where ε is the permittivity of dielectric media in PNSs). Dependence of (**c**) near-field intensity and (**d**) transmittance spectra on the refractive index with dielectric media in the range of 1.0–3.3 in case 2 with *t* = 9 nm. (**e**) The *x*-*z* sectional plane E-field intensity distribution (left) and half-body surface charge density distribution (right) for case 2 (*t* = 9 nm) with *ε* = 3.1, showing selected wavelengths (340, 420, 510, and 770 nm) chosen as representatives of the modes 1–4.

The SPR effect becomes stronger for thinner shell thickness^42^. To validate the relation between the near-field intensity and the transmittance spectra, we only change the thickness of the Ag-shell in case 2 (i.e., *t* = 10 nm replaced by *t* = 9 nm), and kept the other parameters the same. The calculated results of optical performance for t < 9 nm cases (e.g., *t* = 5, 6, 7 and 8 nm) have the same trend as that of *t* = 9 nm, which is an optical spectra red-shift with the decreasing *t* (see the supplementary document). Figure 4a and b show the near-field intensity and transmittance spectra of case 2 with *t* = 9 nm and a series of values for ε (ε = 1.0, 3.0, 5.0, 7.0 and 9.0, where *ε* is the permittivity of dielectric media in PNSs). A decrease in the thickness of the Ag-shell in PNS caused the localized SPR peak to red-shift with increased intensity, while the FWHM narrowed. Interestingly, four distinct modes of transmittance dips were found, as indicated in the inset of Fig. 4a and b, instead of only two modes observed for case 2 with *t* = 10 nm in Ag-shell thickness (see Fig. 2a and b). Each of the trajectory in Fig. 4a and b represents a plasmonic waveguide in the nanochannel of the PNSs and these are observed over a range of dielectric parameter (*ε*) in PNSs. The near-field peak position in case 2 with *t* = 9 nm (Fig. 4a) made a red-shift with a stable near-field intensity whenever a higher *ε* was introduced. For the thinner *t* of 9 nm, the hybrid SPR and CPR modes show larger spectral tunabilitiy than that of the thicker one, *t* = 10 nm. This can be explained in the same way as it is for core-shell nanospheres^43^. For the bonding mode (mode 4), the bonding-like mode (mode 3) and the anti-bonding mode (modes 1–2) with respect to the corresponding transmittance dip, remarkable red-shifts are observed when *t* is reduced and *ε* in PNSs is increased (Fig. 4b). These results indicate the proposed PNSs consisting of larger cores with a thin Ag-shell nanorod show higher tunabilities across the visible and near infrared regions. The observed effects of the inner/surroundings’ permittivity on the localized SPR and the perpendicular CPR can be tuned in the wavelength range of visible and near-infrared. This is highly applicable in photo luminescence, imaging, biomolecule sensing, and photothermal therapy^44^.

We now examine the tunability of the localized SPRs and CPRs on the near-field intensity and transmittance spectrum of case 2 (*t* = 9 nm) for *ε* (the permittivity of dielectric media in PNSs) range of 1.0–3.3, as shown in Fig. 4c and d, respectively. The near-field intensity and transmittance spectrum corresponding to mode 4 are also marked by the green area, as shown in Fig. 4a–d. In Fig. 4c and d, the λ_res_ is sensitive to *ε* in case 2 (*t* = 9 nm), which redshifts from 620 nm to 885 nm as *ε* increases from 1.0 to 3.3. This finding can anticipate that the red-shift can be fully spread and tune in the biological optical window by the variances of *ε*.

To achieve optimal performance for each of the proposed application of the PNSs, the SPs properties such as λ_res_, the E-field intensity, and the bonding mode resonance should all be tailored. Evidently, the cavity in Ag-shell nanorods filled with dielectric medium offers the mechanism to store EM wave energy, and this leads to a subwavelength optical holding with low mode losses^25,38^. This can be further demonstrated by calculating the E-field intensity and charge density in more detail. To better elucidate these findings, we plotted in Fig. 4e the *x*-*z* sectional plane E-field intensity distribution (left) and the half-body surface charge density distribution (right) on the case 2 (*t* = 9 nm) with *ε* = 3.1 for four different wavelengths (340, 420, 510, and 770 nm) chosen as representatives of the modes 1–4, as indicated in the inset of Fig. 4 c and d. According to our previous literature, the longer metal nanorod will produce more standing waves on the metal nanorod^45,46^. When the height (or length) of the proposed PNS is much shorter (i.e., *h* = 150 nm) than that of the incident EM wave, it is difficult to form more standing waves on the surface of the PNS, and the near-field distribution on the surface of the PNS originates mainly from the evanescent wave that propagates in a direction parallel to the interface between the metal and the dielectric medium.

Recognizing the hybridized SP resonance remains a challenge because of the strong mixing of the multiple resonance modes. Here we shall further devote our effort to clarify the derivations of the resonance modes found in case 2 with *t* = 9 nm. As can be seen in Fig. 4e, the near-field pattern is a transition process of the surface charge distribution of the PNSs (see the half-body surface charge density distribution of modes 1–4). At shorter wavelengths of λ = 340 nm (high-order resonances corresponding to mode 1), the PNS exhibits a profile with three distinct sections (i.e., at top, middle and bottom surface of PNS). The E-field resonates on the outer surface of the PNS but with no E-field (i.e. no CPR mode) on the inner surface (or nanochannel) of the PNS. The condition is below the Abbe diffraction limit. At λ = 420 nm, a profile with three sections of E-field resonance is observed (Mode 2). There are distributed on the top, middle and bottom part of nanochannel in the PNS. The E-field resonance appears on the inner surface of the PNS, while the E-field intensity of the outer surface is stronger than the one found on the inner surface. On the contrary, at intermediate wavelengths (λ = 510 nm, mode 3, bonding-like mode), the E-field occurs on both the inner and outer surfaces, and resonates fully inside the nanochannel of the PNS. The E-field intensity of the inner surface is stronger than that of the outer surface. Interestingly, at longer wavelength (λ = 770 nm, mode 4, bonding mode) the E-fields are strongly confined to both the inside and outside the nanochannel of the PNS and occurs far below the Abbe diffraction limit, thus producing a 3D plasmonic nanocavity^38^. They must experience the electric field action in/on the surface of PNSs and the sensitivity can be increased by increasing the surface area and the local E-field enhancement occurs at the PNSs.

**Figure 5:**
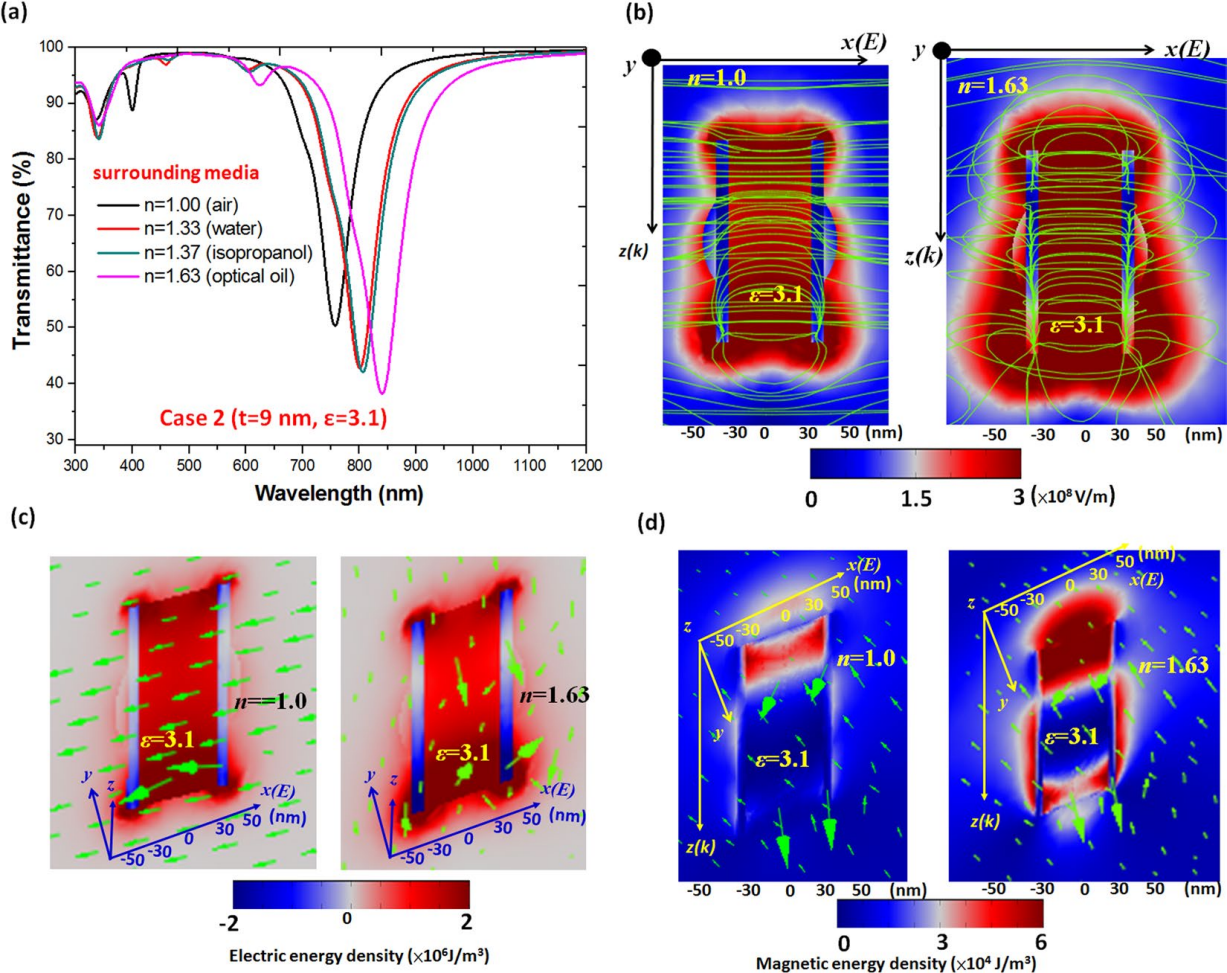
(**a**) Transmittance spectra of case 2 (*t* = 9 nm) with *ε* = 3.1 exposed to air as well as three other fluids (i.e., water (*n* = 1.33), isopropanol (*n* = 1.37) and optical oil (*n* = 1.63)). Comparison of (**b**) E-field intensities (including electric force lines, green lines), (**c**) electric energy density (including electric energy flows, green arrows), and (**d**) magnetic energy density (including magnetic energy flows, green arrows) of the surrounding medium in the case of *t* = 9 nm exposed to air (*n* = 1.0, left panel) and optical oil (*n* = 1.63, right panel).

If the PNSs show stronger SP hybridization in its spectrum, the λ_res_ peak position will shift, depending on the coupling E-field intensities and the electric/magnetic energy flows between the SPRs and CPRs of the inner/outer surface of PNSs. Further analysis examined the influence of the surrounding medium in case 2 (*t* = 9 nm) exposed to air and fluid. In addition to air (*n* = 1), three other fluids with different RIs were used, i.e., water (*n* = 1.33), isopropanol (*n* = 1.37) and optical oil (*n* = 1.63). The transmittance dip position made a red-shift with a slight increase in dip depth whenever a fluid of higher RI was introduced into the PNSs (Fig. 5a). With the increase of RI, a red-shift of the transmittance dip position varied from 760 to 840 nm for case 2 (*t* = 9 nm) with *ε* = 3.1. When the fluid (or molecular) system is resonant with the PNSs, the transmittance spectrum is strongly modified, showing characteristics of strong exciton-plasmon coupling and leading to a bonding resonant mode. This can be examined by the performance of E-field intensity (including the electric force lines (green lines), see Fig. 5b), electric energy density (including electric energy flows (green arrows), see Fig. 5c), and magnetic energy density (including magnetic energy flows (green arrows), see Fig. 5d). In Fig. 5b–d, the presence of resonant fluids (e.g., *n* = 1.00 and 1.63 are selected for comparison) around the PNSs. The presence of fluids affect not only the fluid-field interaction, but also the spatial distribution of the E-field intensity and the electric and magnetic energy flows (time average, J/m^3^) across the junction in the PNSs^47^. In relation to the electric force lines and the energy flow arrows, as shown in Fig. 5b,c, it raises a strong EM field localization and enhancement as well as the energy flows in the dielectric core region (e.g., ε = 3.1) on the inner surface of the PNS far from the metallic surfaces of the PNS^6^. As a result, the effect of the perpendicular CPR arising from the inner surface of the PNS is stronger than that of the outer surface of the PNS, and the cavity resonance in nanochannel is highly sensitive to the changes in RI. The electric force lines and energy flow arrows show a quite different pattern before and after the surrounding medium is introduced into the PNS system. As can be seen in the near-field zone the PNS exposed to a surrounding fluid (optical oil) resulted in a closed form of electric force lines (right panel of Fig. 5b). A loose form of electric force lines is observed when the surrounding medium is air (left panel of Fig. 5b), and when submerged in optical oil a remarkable sensor activity can be found at the interface between PNS and fluid. This can be verified by the starting and ending points of electric force lines in the vicinity of the PNS with a non-air surrounding medium. In the same manner, the electric and magnetic energy flows show an irregular pattern due to the strong interaction between PNS and fluid (right panels of Fig. 5c and d) and the electric and magnetic filed energy flow lines are regular for ε = 1.0, and there is less interaction between the PNS and air (left panels of Fig. 5c and d).

**Figure 6:**
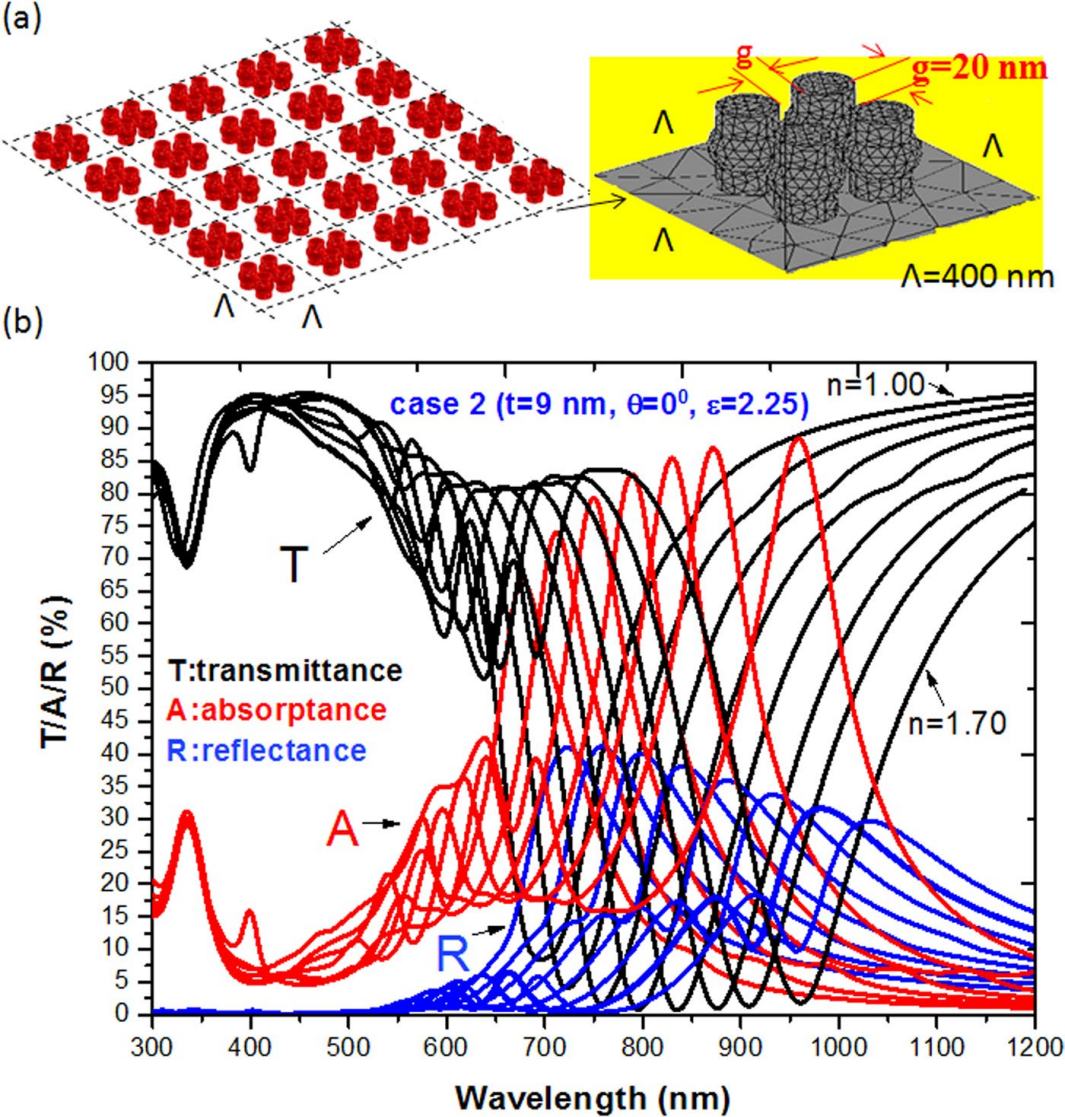
(**a**) A truncated diagram of case 2 (*t* = 9 nm, ε = 2.25, left panel) in the PNS array with 4 × 4 PNSs in a unit cell (right panel). The gap among the PNSs is set to be g = 20 nm. The other parameters are the same as those used in Fig. 4. (**b**) Transmittance (T), absorptance (A) and reflectance (R) spectra of case 2 (*t* = 9 nm) with *ε* = 2.25 exposed to air (*n* = 1.00) and a surrounding medium ranging in 1.00 < *n* < 1.70 in step of 0.1.

**Figure 7:**
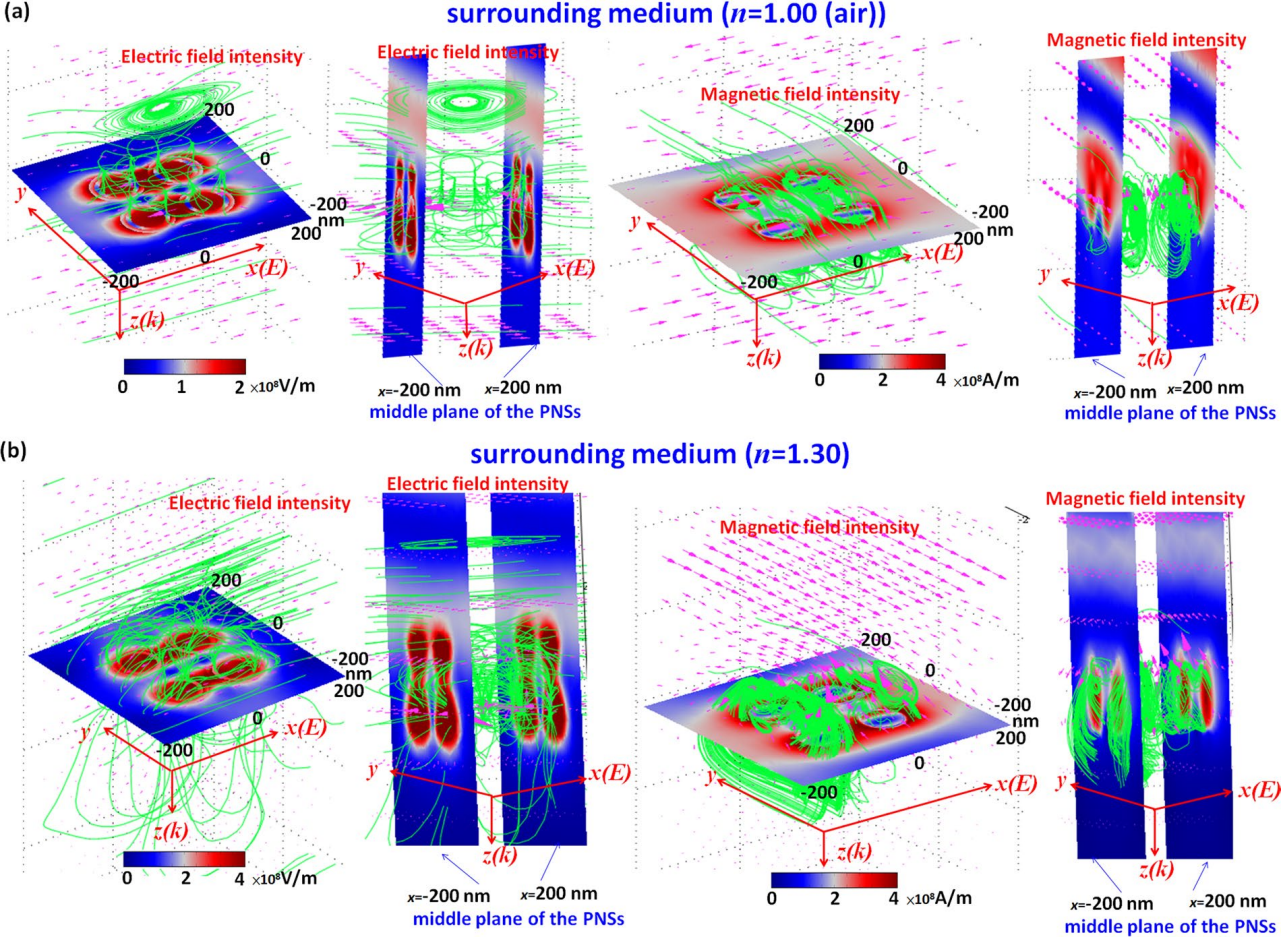
The comparison of E-field intensity and magnetic field intensity (including the 3-D profiles of electric/magnetic force lines (green lines) and energy flows (pink arrows) of case 2 (*t* = 9 nm, ε = 2.25) exposed to (**a**) air (*n* = 1.00) at λ_res_ = 690 nm, and (**b**) a surrounding medium (n = 1.30) at λ_res_ = 800 nm.

In the PNS arrays, light diffraction can mediate the EM waves coupling between the SPRs and CPRs in the neighboring PNSs. Basically, the contribution of SPRs and CPRs in PNS arrays can be further increased since there is more coupling effects among PNSs by introducing more number of PNSs in a unit cell or by reducing the period of array (Λ)^17^. Figure 6a shows the truncate diagram of case 2 (*t* = 9 nm, ε = 2.25, left panel) in the PNS array with 4 × 4 PNSs in a unit cell (right panel). The gap among the PNSs is set to be g = 20 nm and the other parameters are the same as those used in Fig. 4. To examine its performance, the surrounding refractive index (*n*) is changed from 1.00 to 1.70 in step of 0.1. Figure 6b shows the comparison of transmittance (T), absorptance (A) and reflectance (R) spectra of case 2 (*t* = 9 nm) with *ε* = 2.25 exposed to air (*n* = 1.00) and a surrounding medium (1.1 < n < 1.70, in step of 0.1). The spectral absorptance (A) is defined by 1-T-R. The strong coupling of SP modes associated with the dielectric-core and Ag-shell nanorod results in multiple and broadband SPRs and CPRs in the PNSs. Note that the transmittance dip dramatically descended to the values of T = 8.39% (black line) and T = 0.53% (red line) for the case of *n* = 1.00 at λ_res_ = 690 nm and *n* = 1.30 at λ_res_ = 800 nm, respectively, which are much lower than those of the cases with only one PNS in a unit cell (see Figs 2–5). It is evident from Fig. 6b that a lower transmittance dip accompanies with a higher absorptance (e.g., A = 84.603% in the case of *n* = 1.30) and a lower reflectance (e.g., R = 14.867% in the case of *n* = 1.30). These results demonstrate that the above-mentioned bonding mode resonance occurs at the λ_res_ of the transmittance dips. Figure 7a and b are used to compare the E-field intensity and magnetic field intensity distributions (including the 3-D profiles of electric/magnetic force lines (green lines) and energy flows (pink arrows) of case 2 (*t* = 9 nm, ε = 2.25) exposed to air (*n* = 1.00) and a surrounding medium (*n* = 1.30). A remarkable gap enhancement and localization of E-field and magnetic field intensity distributions can be found along the *x* axis and this is the *x*-polarization of the incident EM field. The electric force lines and energy flow arrows of *n* = 1.30 (Fig. 7b) display a denser profile than that of *n* = 1.00 (Fig. 7a), resulting in stronger interactions among the PNSs. These mode patterns and the novel properties are unattainable in the PNS array with 1 × 1 PNS in a unit cell. Since the PNS array with more pairs of PNSs in a unit cell gives rise to the enhanced SPRs and CPRs coupling effects, and it has a narrow bandwidth and nearly zero transmittance dip around the λ_res_. It is expected to have good sensing sensitivity and tunability.

## Discussion

In conclusion, we have proposed a novel approach to the design of PNSs arrays which can provide simultaneously high tunability, sensitivity and compatibility. The influences of the structure and the material parameters, the density of the PNSs in a unit cell, the SPRs and CPRs on the sensing performance of near-field intensity and the transmittance spectra are investigated by using 3-D FEM. By controlling *ε* (the permittivity of dielectric media) and *t* (Ag-shell thickness) in the PNSs, the plasmonic hybrid modes excited on the PNS array can be manipulated and this leads to support multiple resonance modes which are represented as sharp and deep transmittance dips. This feature is practically unachievable in all-metal or all-dielectric photonic systems. The PNSs investigated here have enabled the integration of structured metallic/dielectric nanosphere and nanorod in a single structure. By controlling the hybridization of the SPR and CPR modes in the proposed PNSs with metallic-dielectric interface, the results show the subwavelength E-field confinement and the control over the tuning and sensitivity of PNSs for sensor applications. We have further shown that the novel PNSs (case 2) consisting of metallic/dielectric nanosphere and nanorod is superior to the cases 1 in the literature. The proposed case 2 PNSs can be operated as a RI SPR sensor with a sensitivity of 500 nm/RIU, as well as, a high tunable feature in the range of UV, visible and near-infrared. In addition, a narrow bandwidth and nearly zero transmittance along with a high absorptance can be achieved by a denser arrays of PNSs arranged in the unit cell. Moreover, new phenomena arising from the interaction of SPs and the specific material characteristics may be found in these PNS systems. The potential for bonding-mode resonance operation in conjunction with deep-subwavelength mode sizes suggests the application the proposed PNSs for both fundamental studies and practical applications in the field of plasmonics.

## Methods

3-D FEM based calculations of EM fields around PNSs were performed using the commercial software package COMSOL Multiphysics^48^ and the convergence order of FEM was performed by using quadratic Lagrange elements^48,49^. To mimic an infinite periodic array, the simulation models were obtained from the unit cell by performing a periodic boundary condition (PBC) along both the *x*- and *y*-axes (the lateral directions of the unit cell), and the anisotropic perfectly matched layers (PML) condition on the top and bottom surfaces of the unit cell. This is to reduce the influence of light reflection. A 3-D meshing with a high density tetrahedral mesh was used in the metallic/dielectric nanospheres and nanorods and the surrounding matrix, where the maximum size of the mesh was fixed to be at least 10 times smaller than the effective wavelength of the incident EM wave. The imaginary dielectric constant of Ag will give a negative damping effect to the local SPRs, and the Ag permittivity data cited in ref.^50^ was used. The near-field intensity evaluated point P is located at a distance of 10 nm from the outmost surface of nanosphere (see the inset of Fig. 1c and d). The scattering parameters (S-parameter) which is given with power quantities were used to calculate the transmittance, reflectance and absorptance. The definition of S-parameter, S_11_ and S_21_ are;

1 $${{\rm{S}}}_{11}={[({\rm{power}}{\rm{reflected}}{\rm{from}}{\rm{port}}{\rm{1}})/({\rm{power}}{\rm{incident}}{\rm{on}}{\rm{port}}1)]}^{1/2}$$

2 $${{\rm{S}}}_{21}={[({\rm{power}}{\rm{delivered}}{\rm{to}}{\rm{port}}{\rm{2}})/({\rm{power}}{\rm{incident}}{\rm{on}}{\rm{port}}{\rm{1}})]}^{1/2}$$

The incident light came from port 1 and port 2 functioned as a receiver plane. The reflectance, transmittance and absorptance were calculated from |S_11_|^2^, |S_21_|^2^ and 1−|S_11_|^2^−|S_21_|^2^, respectively. The relative positions of port 1 and port 2 and the PNS are shown in the inset of Fig. 1b.

The sensitivity of the SPR sensor is defined as S = Δλ/Δ*n*, where Δλ is the corresponding central wavelength shift of the resonant dips, and Δ*n* is the difference of the refractive index.

## Electronic supplementary material

Supplementary information
