# Supplementary material for: Simultaneous realization of high sensing sensitivity and tunability in plasmonic nanostructures arrays
Source: Sci Rep. 2017 Dec 1;7:16817. doi: 10.1038/s41598-017-17024-7 (PMC5711893; doi:10.1038/s41598-017-17024-7)
Supplement: Supplementary file 1 — Supplementary information [file 41598_2017_17024_MOESM1_ESM.pdf]

Supplementary information for

Simultaneous realization of high sensing sensitivity and tunability in plasmonic nanostructures arrays

Yuan-Fong Chou Chau[1*], Chan-Kuang Wang[2], Linfang Shen[3], Chee Ming Lim[1], Hai-Pang Chiang[4, 5 †], Chung-Ting Chou Chao[6], Hung Ji Huang[7], Chun-Ting Lin[7], N. T. R. N. Kumara[1] and Nyuk Yoong Voo[1]

[1]*Centre for Advanced Material and Energy Sciences, Universiti Brunei Darussalam, Tungku Link, Gadong BE1410, Negara Brunei Darussalam*

[2]*Department of Electronic Engineering, Chien Hsin University of Science and Technology, No.229, Jianxing Rd., Zhongli City, Taoyuan County 32097, Taiwan (R.O.C.)*

[3]*Institute of Space Science and Technology, Nanchang University, Nanchang 330031, China*

[4]*Institute of Optoelectronic Sciences, National Taiwan Ocean University, No. 2 Pei-Ning Rd., 202, Keelung,Taiwan*

[5]Institute of Physics, Academia Sinica, Taipei 11529, Taiwan

[6]*Department of Physics, Fu Jen Catholic University, New Taipei City, Taiwan*

[7]*Instrument Technology Research Center, National Applied Research Laboratories, Hsinchu, Taiwan*

Corresponding author: chou.fong@ubd.edu.bn, hpchiang@mail.ntou.edu.tw* †

Section S1: Investigation of changing the thickness (t) of the Ag-shell in case 2

To validate the relation between the near field intensity and the transmittance spectra, we change the thickness (t) of the Ag-shell in case 2 (see Fig. 1). The parameters used are indicated in this figure. The calculated results of optical performance of t<9 nm cases (e.g., *t*=5, 6, 7 and 8 nm, see Fig. 2) show the optical spectra red-shift with the decreasing *t*.

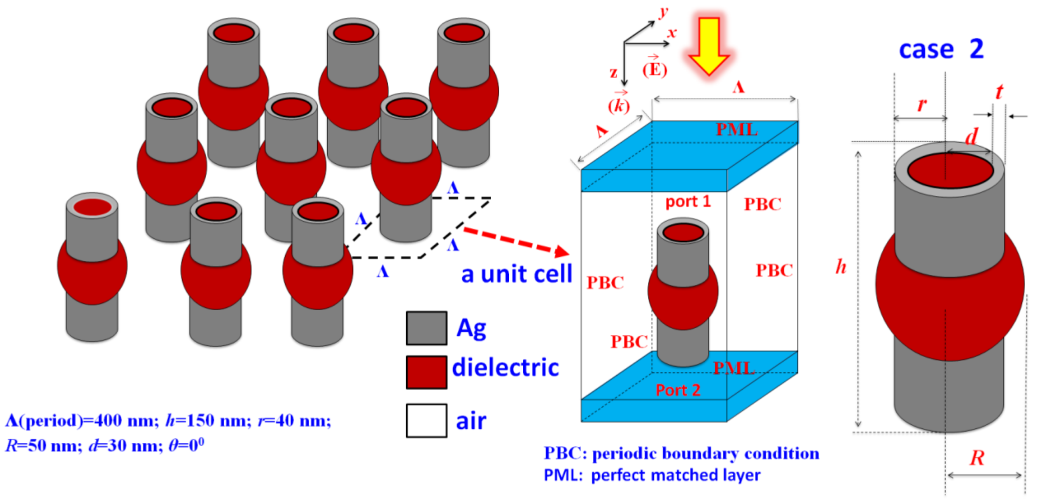

Figure 1 The truncate view of a 2-D periodic array of plasmonic nanostructures (PNSs) (left). The unit cell of the proposed case 2 (middle), case 2 (right): a combination of a metal nanosphere and a core-shell nanorod (metal-shell with a dielectric nanorod in core region). Axis shows the propagation direction and polarization of the incident EM wave. Where the period, height of the nanorod, radius of the nanorod, radius of the nanophere, thickness of the Ag nanoshell and radius of the dielectric nanorod in Ag nanoshell are denoted by Λ, h, r, R, t and d, respectively. The environmental refractive index is set to be 1.0 for air.

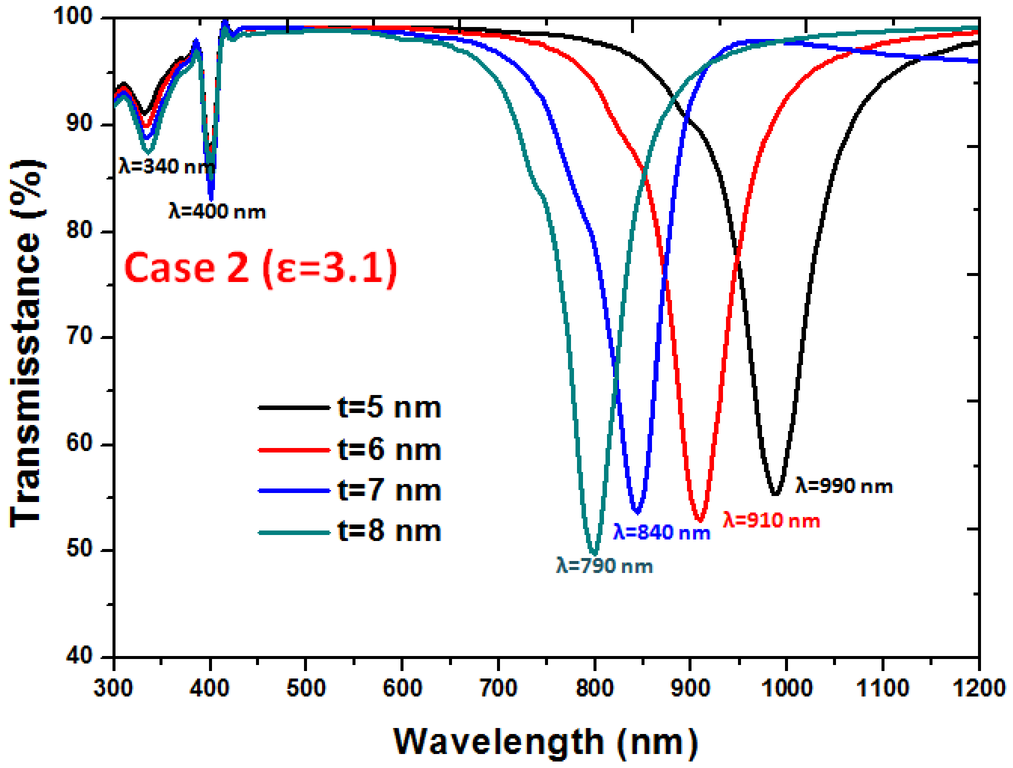

Figure 2 Transmittance spectra of case 2 (ε =3.1) with the shell thickness t<9 nm

(i.e., t=5,6,7 and 9, respectively).

Section S2: Investigation of electromagnetic wave scattering problems

In electromagnetic (EM) wave scattering problems, the total EM wave can be divided into the incident and scattered wave components, i.e., $E=E_{inc}+E_{sca}$ and $H=H_{inc}+H_{sca}$. Important physical quantities can be obtained from the scattered fields. One of these is the cross section, which can be defined as the net rate at which EM energy crosses the surface of an imaginary sphere centered at the particle, divided by the incident irradiation (P_{inc}) (see Fig. 3(a)). To quantify the rate of the EM energy absorbed (W_{abs}) and scattered (W_{sca}) by the particle, the absorption (σ_{abs}), scattering (σ_{sca}), and extinction (σ_{ext}) cross sections are defined as:

$\sigma_{abs}=W_{abs}/ P_{inc}$
$\sigma_{sca}=W_{sca}/ P_{inc}$

where P_{inc} is incident irradiance, defined as energy flux of the incident wave (W/m^2),
W_{abs} is energy rate absorbed by particle (W),
W_{sca} is scattered energy rate (W).
The total scattering cross-section, or extinction cross-section, is the amount of energy removed from the incident EM wave due to absorption and scattering. By energy balance, the extinction cross-section is a superposition of the scattering and absorption cross-sections:

$\sigma_{ext}=s_{abs}+ s_{sca}\,(m^2)$

The total absorbed energy is derived by integrating the energy loss over the volume of the particle. The scattered energy is derived by integrating the Poynting vector over an imaginary sphere around the particle.
Simulations are performed by using a commercial software, COMSOL Multiphysics [1]. It solves for the scattering off of a single structure of case 1 (see Fig. 3(b)) and case 2 (see Fig. 3(c)). The model geometry is shown in Fig. 3(d). Two symmetry planes enable modelling only one quarter of the sphere. The air domain is truncated by a perfectly matched layer (PML), and far-field calculations are done on the inner boundary of the PML domain. The surface S is used to calculate total scattered energy. An incident plane EM wave travels in the positive x direction, with the electric field polarized along the z-axis. This imposes the following boundary conditions on the symmetry planes [2]:

Symmetry in electric fields:

$n \times H|_{y=0}=0$ (Perfect magnetic conductor (PMC))

Symmetry in magnetic fields:

$n \times E|_{z=0}=0$ (perfect electric conductor (PEC))

For the scattered field formulation, Equation ($E=E_{inc}+E_{sca}$) and equation ($\nabla\times(1/\mu_r \nabla \times E_{sac})-k_0^2(\varepsilon_r-j\sigma/\omega\varepsilon_0)E_{sca}=0$)) are used in the model setup. The background incident plane wave is defined as: $E_{inc}=\{0,0,E_0^{e-jk_0x}\}$

where E_0 is plane wave amplitude, $k_0=\omega/c$ is wavevector in the air, ω is circular frequency and c is speed of light in air.

The incident energy flux amplitude can be expressed as $P_0=|P_{inc}|=c\varepsilon_0E_0^2/2$.

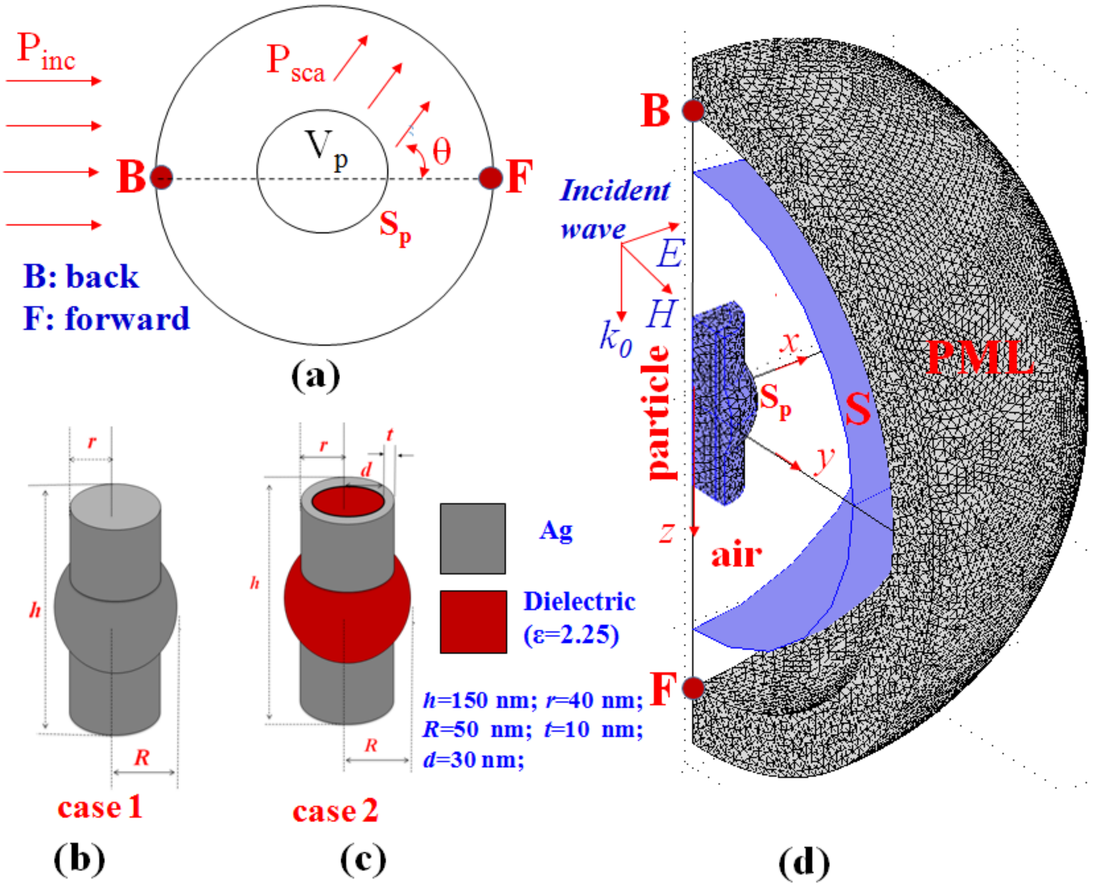

Figure 3 (a) Imaginary sphere S around a particle volume V_p enclosed by a surface S_p. Scattering amplitude is evaluated in the forward direction at point F. Model geometry for a single structure of (b) case 1 and (c) case 2 with $\varepsilon = 2.25$. (d) Generic scattering geometry. An incoming plane wave e^{ikx} scattered off a particle (one quarter of the simulation domain). A great distance away scattered EM wave is approximated by a spherical wave .

The air domain is truncated by a perfectly matched layer (PML) inserted to limit the extent of the simulation model to a manageable region of interest. This PML layer absorbs all outgoing wave energy without any impedance mismatch that could cause spurious reflections at the boundary. The far-field calculations are done on the inner boundary of the PML domain where the near field is integrated. The surface S is used to calculate total scattered energy. An incident plane wave travels in the positive *x*-direction (see Fig. 3(d)), with the electric field polarized along the *z*-axis. Perfect magnetic conductor (PMC) and perfect electric conductor (PEC) boundary conditions are used on the *x*-*z* and *x*-*y* symmetry planes, respectively. The plane EM wave incident on the particle (case 1 or case 2 with ε = 2.25) is defined by its amplitude, wave vector in the air, and circular frequency. Scattering characteristics of the far field spectra, i.e., absorption cross-section, total extinction cross-section near-field calculation and radar cross-section for the case 1 and case 2 considered are shown in Figs. 3(a)-(c), respectively. In addition, the calculated scattering electric field (norm) and scattering magnetic near-field (norm) intensity map of case 1 and case 2 with ε = 2.25 at the prominent resonance wavelengths are also shown in Figs.5 (a) and (b), respectively.

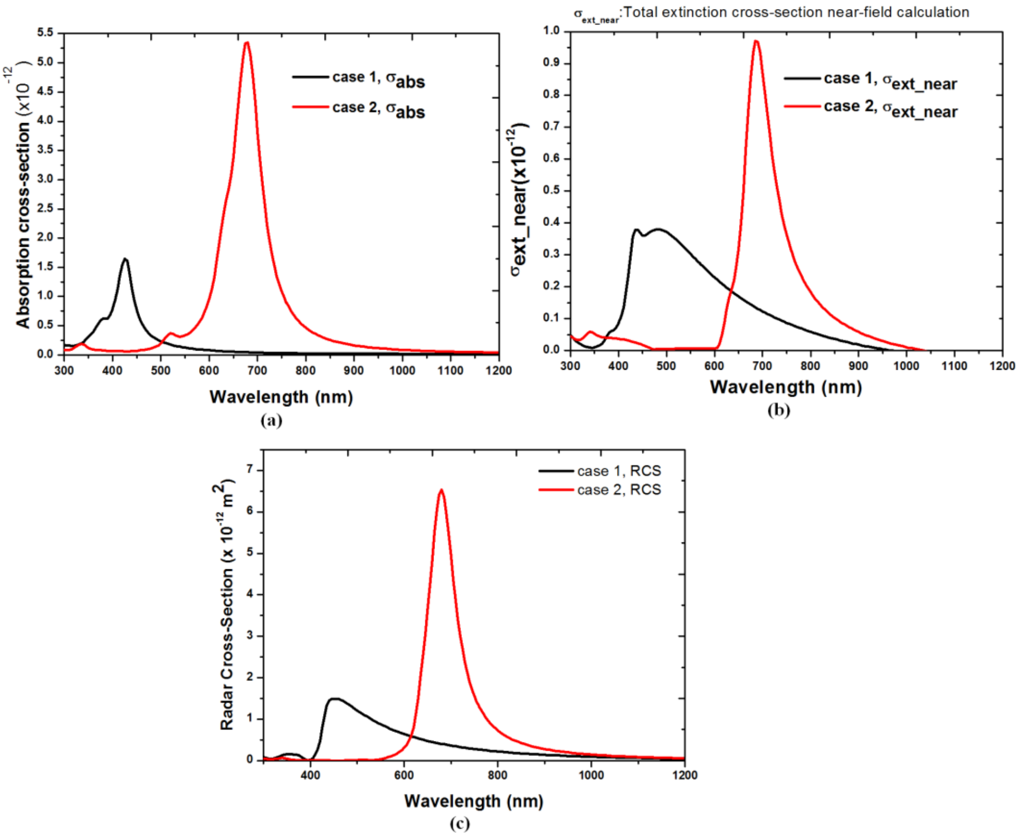

Figure 4 Scattering characteristics of the far field spectra, i.e., (a) absorption cross-section, (b) total extinction cross-section near-field calculation and (c) radar

cross-section for the case 1 and case 2 with $\varepsilon = 2.25$.

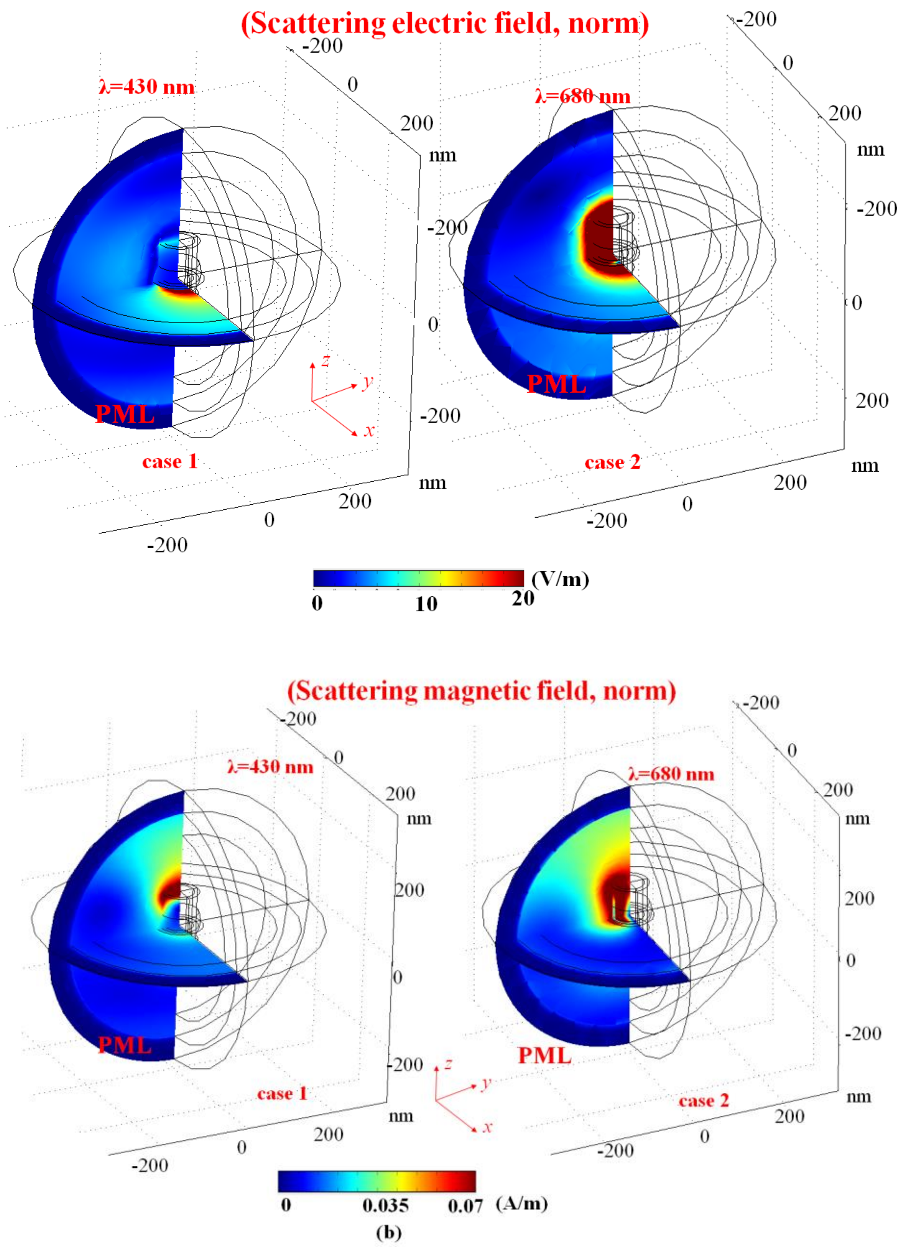

Figure 5 Calculated (a) scattering electric field (norm) and (b) scattering magnetic field (norm) intensity map of case 1 and case 2 with ε = 2.25 at the prominent resonance wavelengths (one quarter of the simulation domain).

Reference

1. https://www.comsol.com/

2. Sergei Yushanov, Jeffrey S. Crompton*, and Kyle C. Koppenhoefer, " Mie Scattering of Electromagnetic Waves", Proceeding of the 2013 COMSOL conference in Boston.
